# The Use of a Sheathless Basket via Miniature Ureteroscopes

**DOI:** 10.1155/DTE.1.177

**Published:** 1995

**Authors:** Graham Watson

**Affiliations:** 1 Consultant Urologist and Senior Lecturer Institute of Urology and St. Peter's Hospital London UK; 2 Department of Urology Institute of Urology and Whittington Hospital 114 Dukes Ave. London N10 2QB UK

## Abstract

The conventional use of a basket involves a metallic wire section that is contained within a polythene sheath. The wire basket can be withdrawn or protruded from the sheath using a handle. The advantage of this system is that the basket can be kept shut or open at will within the ureter. Thus one can keep the basket closed in order to maneuver the basket past a stone before opening it above the stone. With the development of efficient and safe lithotripsy modalities becoming widely available and with miniaturization of these and the ureteroscopes, it is becoming less common to dilate the ureter. Therefore it is not frequently possible to extract a stone intact. Baskets still can be used secondarily to extract the fragments. It is possible to use the basket wire without its sheath. The ureteroscope channel effectively becomes the sheath, and the basket opens immediately on protruding it beyond the ureteroscope. This has the following advantages: (*a*) A larger-diameter basket wire can be introduced. The very small baskets of 2F and 3F used via miniature ureteroscopes are very flimsy. When the sheath is removed, it becomes possible to use an otherwise 3F basket via a 2F channel. (*b*)The operator can work the basket usingjust two fingers close to the instrument channel port. This is more ergonomic than working an opening mechanism at the end of the basket, because this latter system takes the operator's hand away from the instrument. (*c*) The basket can be detached more readily from the ureteroscope, should it become, impacted together with a stone within the ureter. This makes it possible to reinsert the ureteroscope alongside the basket and fragment the stone within the basket, provided that there is no reinforced section to the inner wire at the handle.


*Diagnostic and Therapeutic Endoscopy*, 1995, Vol. 1, pp. 177–181
Reprints available directly from the publisher
Photocopying permitted by license only

© 1995 Harwood Academic Publishers GmbH
Printed in Malaysia

### The Use of a Sheathless Basket via Miniature Ureteroscopes

GRAHAM WATSON

*Consultant Urologist and Senior Lecturer Institute of Urology and St. Peter's Hospital, London*

*(Received February 21, 1994; in final form, August 15, 1994)*

The conventional use of a basket involves a metallic wire section that is contained within a polythene sheath. The wire basket can be withdrawn or protruded from the sheath using a handle. The advantage of this system is that the basket can be kept shut or open at will within the ureter. Thus one can keep the basket closed in order to maneuver the basket past a stone before opening it above the stone. With the development of efficient and safe lithotripsy modalities becoming widely available and with miniaturization of these and the ureteroscopes, it is becoming less common to dilate the ureter. Therefore it is not frequently possible to extract a stone intact. Baskets still can be used secondarily to extract the fragments. It is possible to use the basket wire without its sheath. The ureteroscope channel effectively becomes the sheath, and the basket opens immediately on protruding it beyond the ureteroscope. This has the following advantages: (*a*) A larger-diameter basket wire can be introduced. The very small baskets of 2F and 3F used via miniature ureteroscopes are very flimsy. When the sheath is removed, it becomes possible to use an otherwise 3F basket via a 2F channel. (*b*) The operator can work the basket using just two fingers close to the instrument channel port. This is more ergonomic than working an opening mechanism at the end of the basket, because this latter system takes the operator's hand away from the instrument. (*c*) The basket can be detached more readily from the ureteroscope, should it become, impacted together with a stone within the ureter. This makes it possible to reinsert the ureteroscope alongside the basket and fragment the stone within the basket, provided that there is no reinforced section to the inner wire at the handle.

**KEY WORDS:** Sheathless basket, ureteric calculus, ureteroscopy, residual fragments

### INTRODUCTION

The range of instruments to extract calculi is a testimony to the ingenuity of urologists of the 19th and 20th centuries. Balloons were used to dilate sections of the ureter below stones (1, 2). Multiple catheters were passed alongside stones to overcome the obstructing epithelial edema (3). Loops were constructed from lengths of suture and ureteric catheter (4, 5). Finally baskets were designed to catch the stone by engaging it within a wire cage (6, 7). With the greater ability to engage the stone and exert traction came a potential for trauma to the ureter from traction injury (8). With the advent of ureteroscopy, it became possible to engauge the stone under vision, but the potential for ureteric trauma was maintained. The ureter could be damaged by the passage of the ureteroscope or by the forced extraction of the stone. Modern techniques of stone fragmentation allow the stone to be fragmented under vision rather than extracted intact. The fragmentation of a stone avoids the risk of extracting a stone too large for the ureter. However, there is in its place the problem of local trauma from the stone fragmentation and that of elimination of the stone fragments. Modern miniaturized ureteroscopes have reduced the trauma associated with insertion of the ureteroscope. These instruments do not require dilatation of the distal ureter. However, this in turn reduces the ability to extract the calculus. Modern miniaturized lithotripsy systems such as the lithoclast (9) and the pulsed dye laser (10) have contributed to the urologist's armamentarium by reducing the trauma from the stone fragmentation.

Most urologists fragment the stone and leave the fragments to pass spontaneously. Watson et al (11) conducted a retrospective audit of cases managed in this way versus cases where a basket was used to extract as many fragments as possible. There were 139 calculi where the stones were fragmented and the fragments left to pass spontaneously. There were 74 calculi where a basket was used to extract as many fragments as possible. In the group where the fragments were left to pass spontaneously, 72% of cases

Address for correspondence: Graham Watson, M.D., Department of Urology, Institute of Urology and Whittington Hospital, 114 Dukes Ave., London N10 2QB, England.

were cleared eventually by a single ureteroscopy alone. This left 15% that required a second ureteroscopy, 2.2% that required a third ureteroscopy, 9.5% that required ESWL to fragments flushed to the kidney, and 1.5% that required percutaneous nephrolithotomy. In the group treated by fragmentation plus basketing of residual fragments, 89% were cleared by a single ureteroscopy. This left 9% where a second ureteroscopy was required, and 1% where follow-up ESWL was required. Residual stone fragments were found to clear very gradually from the ureter. In the group that was not treated with a basket, there were 32 patients who cleared their fragments prior to discharge, and 67 patients who passed the fragments subsequently. Of these 67 patients, 18 took 1 month, 28 took between 1 and 3 months, and 21 took more than 3 months. Thus the use of a basket to clear the ureter results in a lower reoperation rate and a shorter time before the patient is rendered stone free. The miniaturized ureteroscope allows multiple reentries into the ureter to withdraw one or more fragments until the ureter is free of visible fragments. This now has become the standard strategy in our department. The only exception to this strategy is when there is difficult access to the stone or if any trauma to the ureter occurs. In these cases a stent is placed, and the patient is observed for spontaneous elimination of fragments.

### METHOD

The technique of insertion of the basket and controlling the opening and closing of the basket is shown in Figures 1 through 5, where the example shown is a Candela MiniSope and a Segura flat wire basket. A 3F Segura flat wire basket (Microvasive Corp., Watertown, Ma) has a central metal section of approximately 2F, which can be used via the 2.2F channel of the MiniScope (Candela Corp., Wayland, Ma). The handle of this basket is detached, and the sheath is removed leaving only the 2F metallic basket. The basket section is compressed between finger and thumb so that it can be inserted into one of the instrument channels (Fig. 1). This is made easier by leaving a 3-cm length of sheath over the basket section. This small section of sheath acts as an introducer for the basket into the ureteroscope. As soon as the basket emerges from the metallic instrument channel of the ureteroscope, it starts to open in exactly the same manner as it would when protruded from the sheath (Fig. 2). Pulling on the wire gives controlled closure of the basket (Figs. 3 and 4). When a stone fragment is caught in the basket, it can be held more tightly by gentle traction on the basket, which closes down against the end of the instrument channel. There is no risk to the optics of the

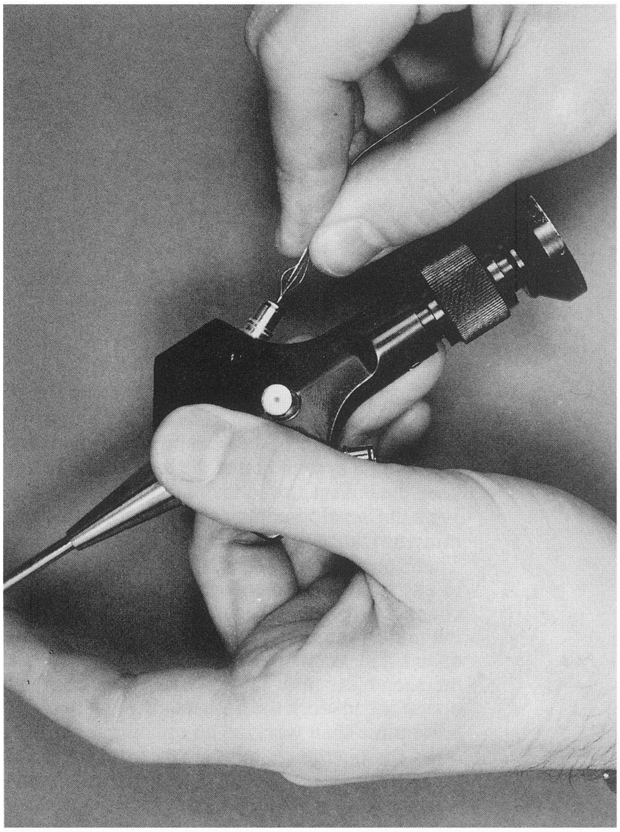

**Figure 1** The wires of the basket are compressed to insert the basket into the instrument channel.

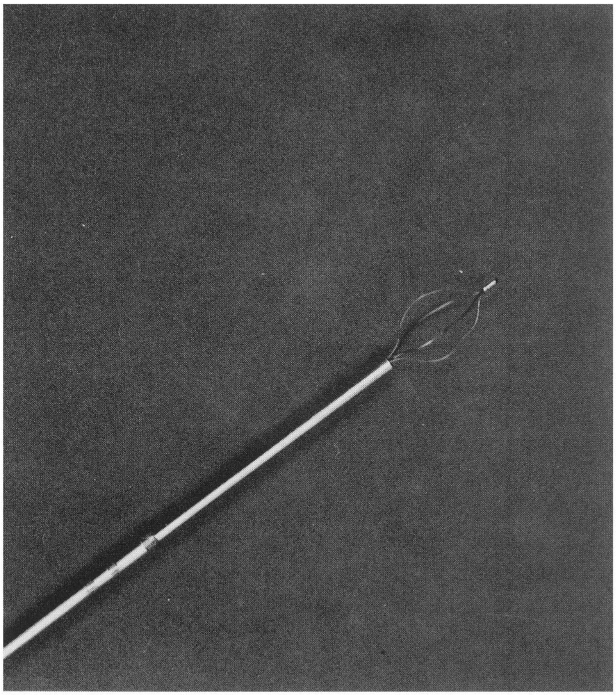

**Figure 2** The basket opens completely when emerging from the ureteroscope.

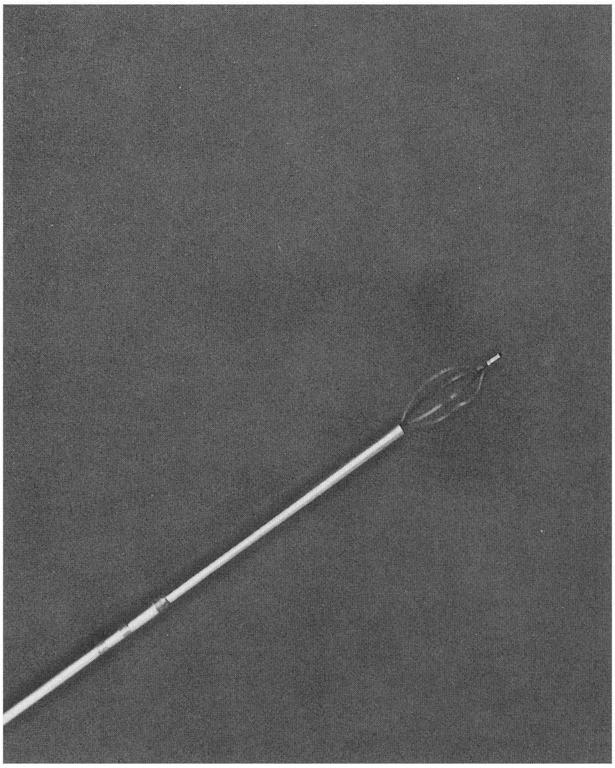

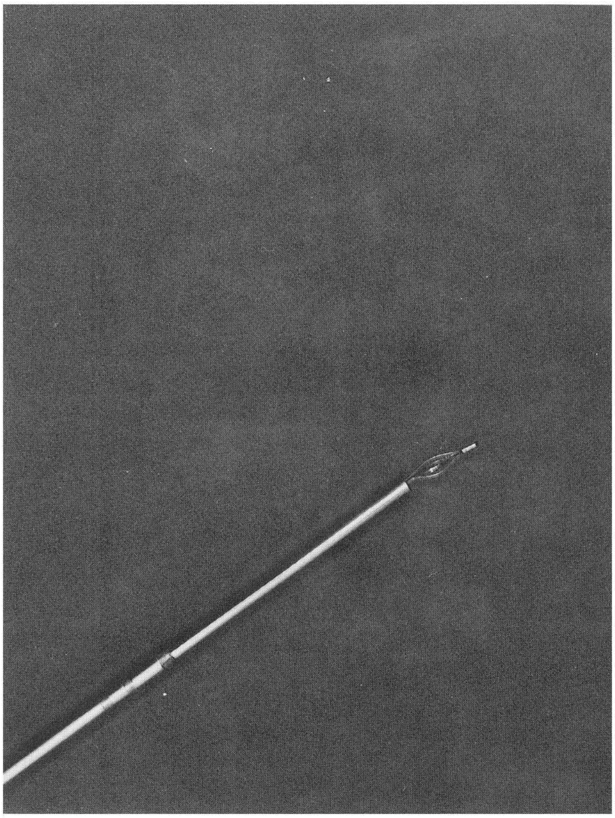

**Figure 3 and 4** Controlled closure of the basket is acheived while withdrawing it into the instrument channel.

ureteroscope. The basket can be controlled completely using just two fingers of the hand that continues to hold the ureteroscope. This leaves the other hand completely free for example for applying abdominal pressure during a difficult ureteroscopy in order to straighten the ureter ahead. If a fragment proves too large to extract, it starts to impact in the ureter with the basket around it. The basket can be disengaged from the ureteroscope very easily, provided there is no reinforcing to the handle section of the wire, simply by withdrawing the ureteroscope. This leaves the basket with its enclosed stone within the ureter. The ureteroscope then can be reintroduced into the ureter alongside the narrow basket. The stone is reached and fragmented within the wires of the basket. The basket can then be withdrawn. This maneuver is relatively difficult. Further it is theoretically possible to fragment the basket, especially if using electrohydraulic probes or a holmium laser. Therefore ideally the stone is broken down into fragments that can be extracted. When extracting fragments, one should engage the lowest-lying fragments first. Otherwise the basket might become impacted with multiple fragments.

Attempted basket clearance of all residual stone fragments has become our routine whenever using the uretero-

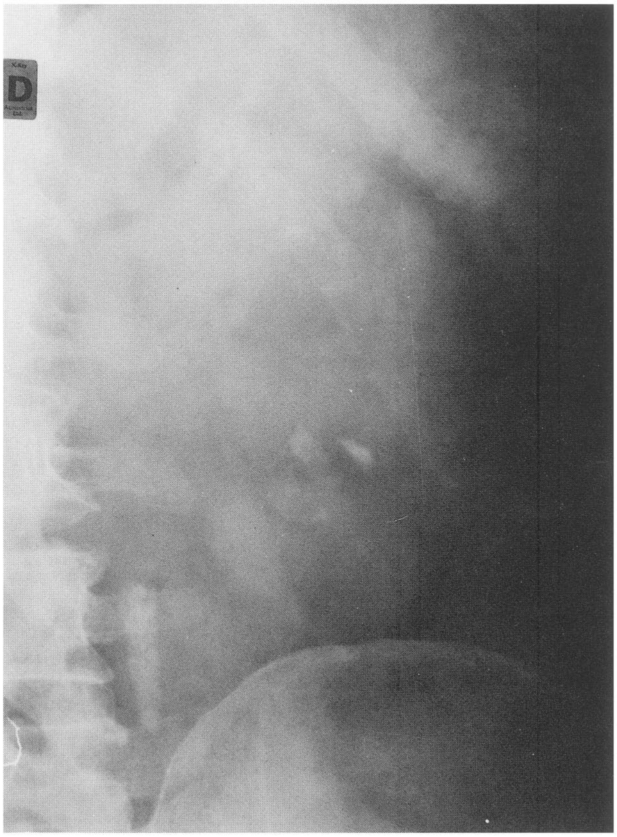

**Figure 5** Preoperative plain x-ray of an upper third steinstrasse.

scope. The endpoint of the operation becomes much clearer; it is when the ureter is free of all fragments. This policy is not adhered to when the ureter is difficult or if any trauma to the ureter occurs during instrumentation or fragmentation. Under these circumstances, a JJ stent is inserted at the first opportunity. Apart from these rare instances, all ureters are cleared at the time of surgery. This is possible even when there is a relatively large stone bulk as in Figure 5. Multiple passes with the ureteroscope and basket were made until the ureter was clear both visually and radiologically. A plain x-ray taken the following morning showed complete clearance of fragments even from the kidney above the obstruction (Fig. 6).

The same technique can be applied in the case of triradiate grasping forceps. When these are pulled against the metallic section of a ureteroscope instrument channel, they close with sufficient strength for removal of a migrated from the ureter (Figs. 7 and 8).

### FUTURE DEVELOPMENTS

Baskets made of nytenol rather than stainless steel have greater memory and durability. The 4F nytenol basket made by Angiomed Corp, Germany, would be suitable for use via 3F instrument channels if they did not have a 4F tip at the end of the basket. This would be a minor modification. The Dretler laser basket of 5F (Cook Urological Inc., Spencer, In,) is a hollow basket to take a laser fiber. The fiber centers on a stone within the basket. The use of a sheathless Dretler basket would allow this system to be used via miniaturized ureteroscopes with their smaller instrument channels. When faced with a highly mobile stone, it may be necessary to immobilize it in a basket. It is possible to use a basket and a fragmentation device simultaneously via a ureteroscope, but this generally demands the use of a larger caliber ureteroscope. We have generally trapped the stone in a basket, withdrawn the ureteroscope leaving the basket and stone in situ, and repassed the ureteroscope as described in the previous section. This extra maneuver would be no longer necessary if a small caliber hollow basket were to be made available. This would be possible if it were sheathless.

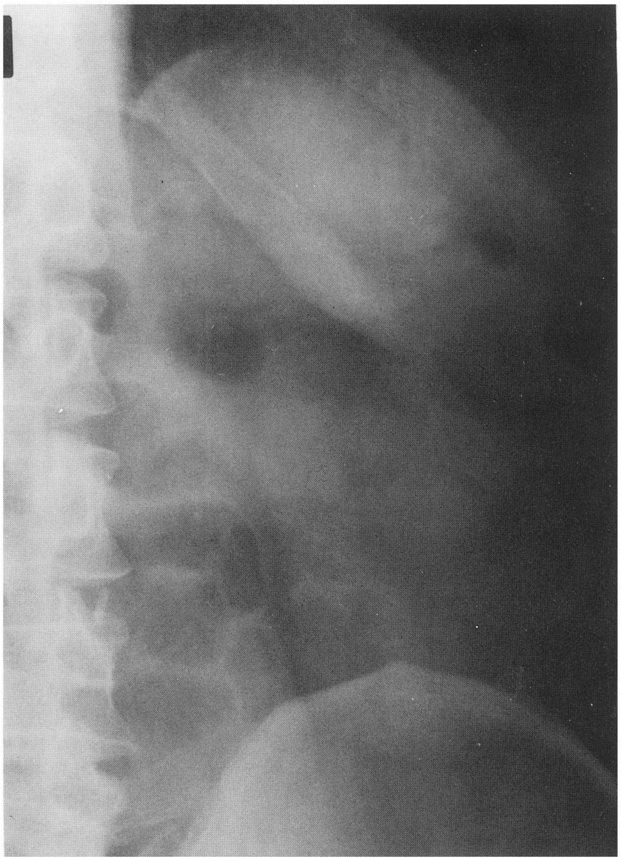

**Figure 6** Plain x-ray at 48 hours postoperatively is free of fragments.

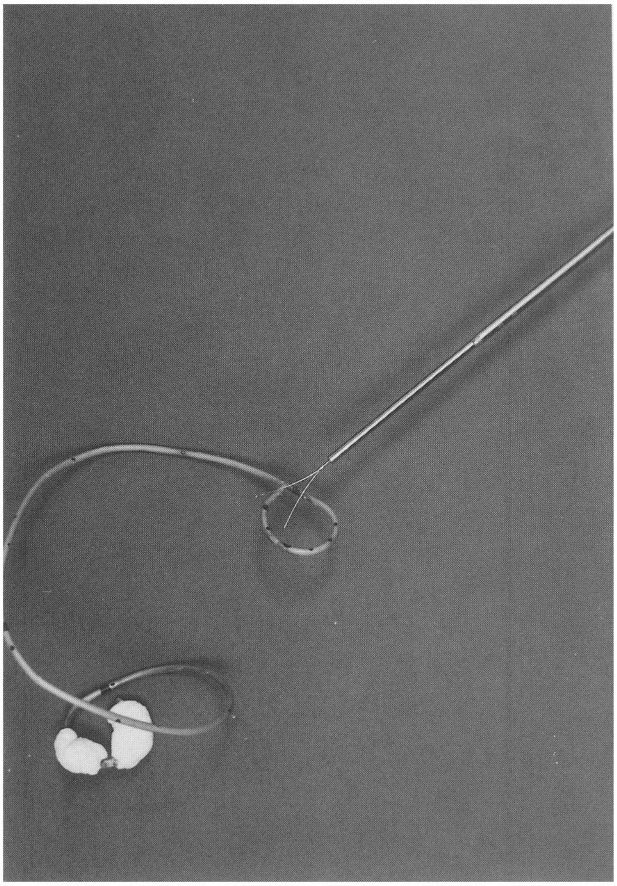

**Figure 7** A sheathless triradiate grabber for grasping a JJ stent.

The advantages of using a basket without a surrounding sheath are that a larger basket can be used for a given in-

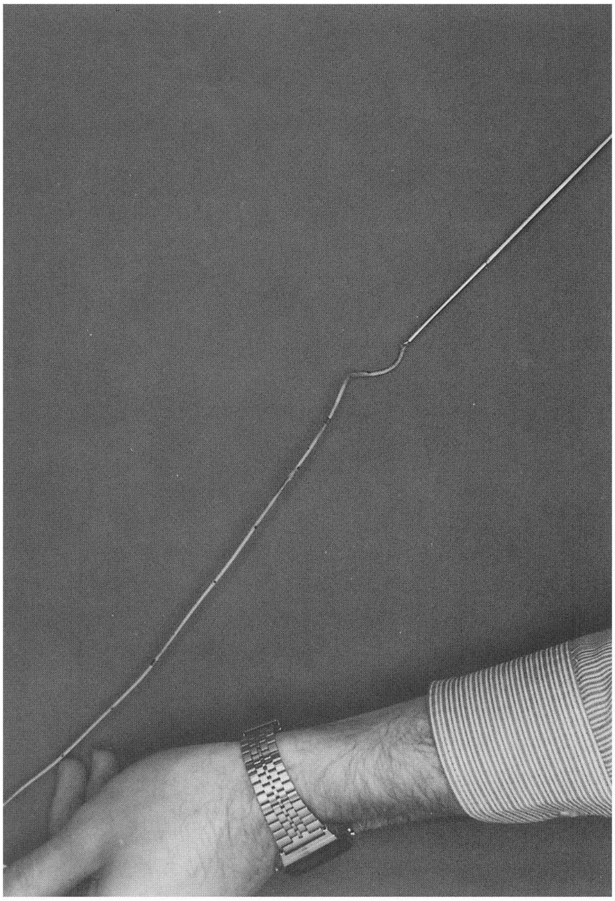

**Figure 8** A firm grip can be made on the stent by withdrawing the grabbers against the instrument channel.

strument channel caliber and that opening and closing the basket can be controlled with just two fingers. This advantage holds only when the manufacturers do not have an expanded "bobble" on the tip of the basket. Also the wire of the basket is sometimes reinforced to take a handle. This can interfere with the maneuver of removing the ureteroscope and reinserting it alongside the basket (as mentioned in the preceeding section). Baskets should be designed for the moment with the ability to be used with or without a sheath.

### REFERENCES

1. Nitze M. Eine neue beobachtungs—und unersuchungsmethode fur harnrohre, harnblase und rectum. Wien Meditzin Wischenschreibe 1879;29:649,688,713,779 and 806.
2. Dourmashkin RL. Cystoscopic treatment of stones in the ureter with special reference to large calculi; based on the study of 1550 cases. J. Urol 1945;54:245–283.
3. Crowell AJ. Removal of ureteral stone by cystoscopic manipulation. J Urol 1921;6:243–255.
4. Zeiss L. Uber eine neue methode der konservativen harnleitersteinbehandlung (die harnleitersteinextraktion mit den schlingenkatheter.) Zeitschrift Urologe 1939;33:121–158.
5. Davis TA. removal of ureteral calculus by new catheter type extractor. J Urology 1954;72:346–349.
6. Councill WA. New ureteral stone extraxtor and dilator. JAMA 1926;86:1907–1908.
7. Dormia E. Dormia basket: standard technique, observations and general concepts. Urology 1982;20:437.
8. Drach GW. Stone manipulation: moderan usage and occasional mishap. Urology 1978;12:286–289.
9. Hofbauer J, Hobarth K, Marberger M, Lithoclast: New and inexpensive mode of intracorporeal lithotripsy. J Endourol 1992;6:429–432.
10. Watson GM, Murray S, Dretler S, Parrish JA. The pulsed dye laser for fragmenting urinary calculi. J Urol 1987;138:195–198.
11. Watson GM, Lander B, Nauth-Misir R, Wickham JEA. Developments in the ureteroscopes, techniques and accessories associated with laser lithotripsy. World J Urol 1993;11:19–25.

